# Total Substitution of Egg White by Faba Bean Protein Concentrate in Marshmallow Formulation

**DOI:** 10.3390/foods15020382

**Published:** 2026-01-21

**Authors:** Ameni Dhieb, Abir Mokni Ghribi, Haifa Sebii, Zina Khaled, Romdhane Karoui, Christophe Blecker, Hamadi Attia, Souhail Besbes

**Affiliations:** 1 Laboratory of Analysis Valorization and Food Safety, National Engineering School of Sfax, University of Sfax, Sfax 3029, Tunisia; amenidhieb1996@gmail.com (A.D.); mokniabir@yahoo.fr (A.M.G.); haifa.sebii@enis.tn (H.S.); zina.khaled@enis.tn (Z.K.); hamadi.attia@gmail.com (H.A.); 2 Highly Institute of Applied Sciences of Mednine, University of Gabes, Mednine 4119, Tunisia; 3 Highly Institute of Biotechnology of Beja, University of Jendouba, Beja 9000, Tunisia; 4 Institut Charles VIOLLETTE, EA 7394, University of Lille, F-59000 Lille, France; romdhane.karoui@univ-artois.fr; 5 Laboratory of Food Science and Formulation, Gembloux Agro-Bio Tech, University of Liège, 5030 Gembloux, Belgium; christophe.blecker@ulg.ac.be

**Keywords:** egg white, faba bean protein concentrate, marshmallow, foaming properties, rheological properties

## Abstract

This paper discusses the total replacement of egg white (EW) with faba bean protein concentrate (FPC) in a marshmallow formulation. The physico-chemical and techno-functional characterizations of the ingredients revealed that FPC, with a protein content of 68%, exhibited an interesting foaming capacity (200%) compared to EW, which had comparable foaming stability. The physico-chemical properties of the final products indicated that the FPC marshmallow (FPCM) had a higher density (0.519 g/mL), lower moisture (17.337%), and a water activity within the recommended range for this type of product. The FPCM had the highest hardness and elasticity values but the lowest cohesiveness and adhesiveness. Scanning electron microscopy showed that the FPCM structure is similar to that of the EW marshmallow (EWM). In front-face fluorescence spectroscopy measurements, the FPCM exhibited higher emission intensity for tryptophan with a maximum at 382 nm and vitamin A with a maximum located around 338 nm. FTIR analysis presented higher peaks at 850, 918, and 1034 cm^−1^ for the EWM compared to the FPCM. In a hedonic evaluation, the majority of descriptors (hardness, odor, and general acceptability) showed similar scores for both formulations. All results demonstrated the success of the total substitution of egg white by FPC in the marshmallow formulation.

## 1. Introduction

Marshmallows are sugar-based confectionery products that are widely consumed across different age groups and populations due to their soft texture and sweet flavor. The air and moisture introduced during the whipping of ingredients give marshmallows their characteristic foamy and soft texture [1]. The main ingredients in marshmallows include gelatin, sugar, glucose syrup, egg white, and flavorings [2]. The production process involves heating a sugar syrup composed of sucrose, glucose syrup, and water to a precise temperature and concentration, which allows for mechanical whipping without the collapse of the foam structure. During whipping, proteins such as egg white migrate between the liquid and gas phases, stabilizing air bubbles and giving marshmallows their characteristic volume and elasticity. The delicate balance of temperature, sugar concentration, and protein functionality represents a major technological challenge in marshmallow production, as deviations can result in syneresis, the collapse of foam, or an undesired texture. Egg white is a key structural component in aerated confectionery systems, where its proteins provide high foaming capacity, foam stability, and viscoelastic interfacial film formation, resulting in a soft and light product texture. Hydrophilic groups migrate to the liquid phase (e.g., syrup), while hydrophobic groups migrate to the gas phase. Whipping brings hydrophobic groups to the bubble surfaces, reducing surface tension and stabilizing the foam. This precise mechanism allows marshmallows to maintain their volume and texture during storage and handling. Although bird eggs are nutritionally rich ingredients containing high-quality proteins, lipids, vitamins, and minerals, their use in food manufacturing raises sustainability-related concerns, including allergenicity, dietary restrictions, and environmental impacts associated with animal-based protein production. Allergies to egg proteins are common in children, and roughly one-third persist into adulthood [3]. Moreover, large-scale egg production is associated with environmental and ethical challenges, including high water and feed requirements, greenhouse gas emissions, and animal welfare concerns. Plant proteins are increasingly investigated as alternative protein sources due to sustainability considerations and dietary diversification. However, their nutritional quality, techno-functional performance, and digestive behavior are highly dependent on protein structure and processing conditions. Indeed, plant proteins may exhibit different gastrointestinal digestibility and functional properties compared with animal proteins, and these differences cannot be generalized without considering the protein source and processing method [4]. Research has demonstrated that plant proteins can provide techno-functional properties comparable to animal proteins, including hydration, emulsification, foaming, and gelation [5]. These qualities make them suitable for incorporation into various food matrices. Legume proteins, in particular, have become popular over the last 15 years due to their ability to fix fat; retain water; dissolve in aqueous systems; and form gels, foams, and emulsions [6,7]. Marshmallows represent an ideal model system for studying protein substitution because their quality is highly dependent on protein functionality, particularly foaming and gelling, making them a sensitive indicator of alternative protein performance.

Faba bean protein has emerged as a promising plant-based substitute for animal proteins, especially in egg-free and dairy-free formulations. Faba bean protein isolates and concentrates exhibit strong emulsifying and foaming properties, which are critical for applications in plant-based cakes, sweets, whipped toppings, and egg-free mayonnaise [6]. Its main protein components, vicilin (7S) and legumin (11S), are responsible for stabilizing air–water and oil–water interfaces, providing functionality comparable to that of egg proteins. In addition, faba bean protein offers a favorable amino acid profile, particularly high lysine levels, and is free of common allergens, making it suitable for vegan, gluten-free, and hypoallergenic diets. Advanced processing methods, such as ultrafiltration, enzymatic hydrolysis, and pH shift treatments, can further improve its solubility, digestibility, and functional properties, enabling broader application in innovative plant-based products.

The replacement of egg white with faba bean protein also addresses growing consumer demand for clean-label, eco-friendly, and sustainable protein sources. By exploring this substitution, it is possible to reduce allergenicity, lower environmental impact, and meet the rising market interest in plant-based confectionery.

This study aimed to investigate the feasibility of replacing egg white with faba bean protein extract in marshmallows. This study involved the physico-chemical and techno-functional characterization of both protein ingredients, followed by the preparation of marshmallows. The resulting products were evaluated in terms of physical and textural properties, and a hedonic assessment was conducted to determine the consumer acceptability of the novel formulation.

## 2. Materials and Methods

### 2.1. Raw Material

Sugar (type S2), egg white (“Everyday” brand), and glucose syrup (38–42 DE) were purchased from a local market in Sfax.

Gelatin powder (bovine 220 g Bloom) and faba bean protein concentrate (FPC) were supplied from the General Society of Food Additives and Adjuvants (SG3A) (Sfax, Tunisia). According to the supplier’s technical data sheet and confirmed by Kjeldahl analysis (Table 1), the protein content of the concentrate ranged between 68 and 70% (*w*/*w*).

### 2.2. Physico-Chemical Properties of Egg White and Faba Bean Protein Concentrate

#### 2.2.1. Moisture Content

The moisture content was determined by drying the samples at 105 °C to a constant weight, in accordance with the Association of Official Analytical Chemists (AOAC, 1995).

#### 2.2.2. Protein Content

The protein content of faba bean protein concentrate (FPC) and egg white (EW) was determined using the conventional Kjeldahl method. Heating in the presence of sulfuric acid (18 mol/L) and a suitable catalyst caused the organic materials to mineralize. After the reaction’s products were alkalized and ammonia was distilled, they were collected in a boric acid solution and titrated with a 0.1 N sulfuric acid solution. The protein content was deduced using the conversion factor (6.25 for faba bean protein concentrate and 6.35 for egg white) (AOAC, 1995).

#### 2.2.3. Water Activity

Water activity (a_w_) was determined at 25 °C, with an instrument (Novasina Aw Sprint TH- 500, Axair Ltd., Pfäffikon, Switzerland). The instrument was calibrated using six certified water activity calibration standards (11%, 33%, 53%, 75%, 90%, and 98% relative humidity) supplied by Novasina (Switzerland) at 25 °C.

#### 2.2.4. pH Values

pH measurements were conducted for faba bean protein concentrate (FPC) dispersions and liquid egg white (EW) using a pH meter (744, Metrohm, Herisau, Switzerland) at 20 °C. The pH of faba bean protein concentrate (FPC) was measured on a dispersion prepared by dissolving 1 g of FPC in 10 mL of distilled water, ensuring a homogeneous solution. For egg white (EW), the pH was measured directly on the fresh material after homogenization.

### 2.3. Techno-Functional Properties of Egg White and Faba Bean Protein Concentrate

#### 2.3.1. Water Holding Capacity

The water holding capacity (WHC) was determined according to McConnell et al. [7]. Prior to WHC determination, the moisture content of the samples was measured and found to be 88.37 ± 0.06% for liquid egg white (EW) and 3.91 ± 0.09% for faba bean protein concentrate (FPC). For both protein sources, the same amount of dry matter (0.5 g) was used. For faba bean protein extract, 500 mg of dry powder was weighed. For egg white protein, the corresponding mass of liquid egg white was calculated based on its moisture content.

Each sample was then diluted to a final volume of 50 mL with distilled water in a volumetric flask, ensuring identical solid concentration and total volume for both proteins.

The mixtures were incubated for 1 h with vortex agitation (15 s) every 15 min, followed by centrifugation at 7155 *g* for 20 min. The supernatant was removed, and WHC was expressed as grams of water retained per gram of dry matter.

#### 2.3.2. Surface Properties

##### Foaming Properties

The techniques defined by Mustafa et al. [8], with slight changes, were utilized to measure foaming capacity (FC) and foaming stability (FS). A 50 mL graduated beaker was filled with 40 mL of the sample (10% of faba bean protein concentrate), which was then whipped for 2 min at 24,000 rpm using an ULTRA-TURAXT25 basic (IKAWERKE, Germany). Before and after homogenization, the volumes were measured, and the following formula was used to determine the rise in volume as a percentage:

(1) $$\mathrm{FC}\;\%=\frac{V2-V1}{V1}.100$$

Here, V2: volume of the solution after whipping; V1: volume of the solution before whipping. The volume of the residual stable foam was compared to the original volume to determine the foam stability (FS) at 25 °C after 30 and 60 min.

##### Surface Charge Measurement

Surface charge measurements was performed on solutions containing 0.1 g of protein per 100 mL at the native pH of each protein. The actual protein content of egg white powder and faba bean protein concentrate was previously determined, and the appropriate amount of each material was accurately calculated and weighed to achieve an equivalent protein concentration (0.1 g protein/100 mL) using a volumetric flask. This approach ensured the full standardization of protein concentration and comparability between the two protein systems.

#### 2.3.3. Gelling Capacity

##### The Least Gelation Concentration (LGC)

Solutions containing 5–20% (*w*/*v*) of faba bean protein concentrate powder, prepared in 1% (*w*/*v*) increments (i.e., 5, 6, 7, …, 20%), were heated at 90 °C for 30 min and then immediately cooled in an ice bath for 20 min [9]. The lowest protein concentration at which the sample did not flow upon the inversion of the tube for 30 s was recorded as the LGC.

##### Texture Profile Analysis

The textural properties (hardness, cohesion, elasticity index, and chewiness) of the EW and the faba bean protein concentrate gels were determined using texture profile analysis. A Texturometer (LLOYD instruments, Fareham, UK) was used to exploit the force–time curve obtained during two successive compression cycles of the gels. Gels were prepared under identical formulation and processing conditions, as described in the Least Gelation Concentration (LGC) Section. Cylindrical gel samples (20 mm height × 25 mm diameter) were stored at 4 °C for 24 h to allow for complete gel setting and equilibrated to 20 ± 1 °C before analysis. The test was carried out using a 35 mm diameter cylindrical probe. The sample was compressed by 50% at a speed of 1 mm/s. The time between two cycles was 5 s. All measurements were carried out in triplicate.

Texture parameters, including hardness, cohesiveness, elasticity (springiness), and chewiness, were automatically calculated from the force–time curves using “Exponent Connect” software (version 7.0).

### 2.4. Preparation of Marshmallow

**Figure 1:**
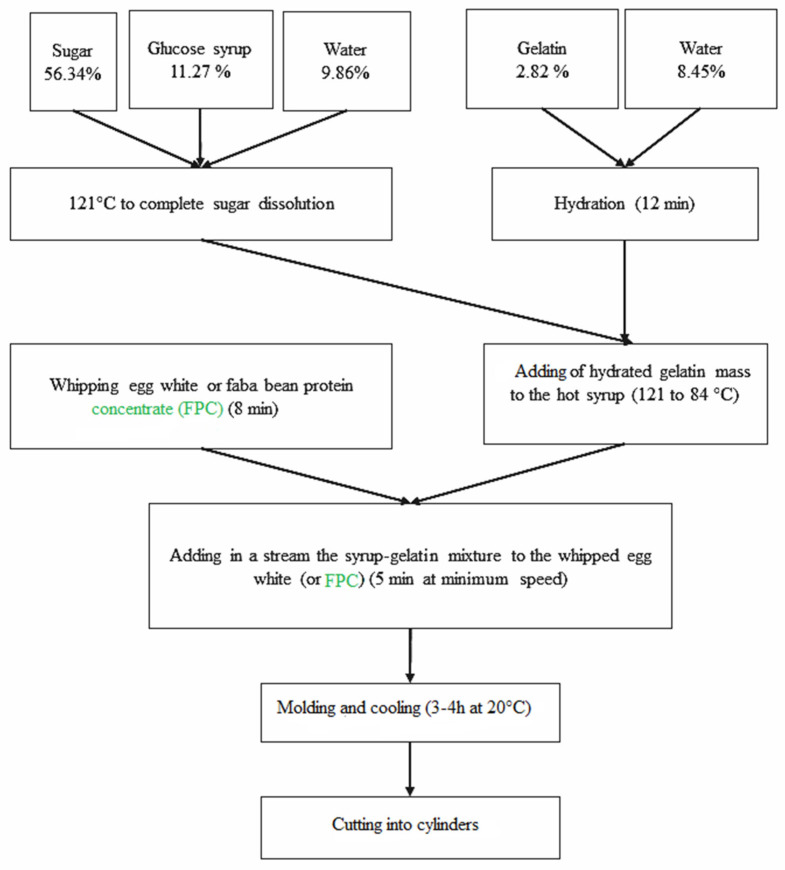
Procedure of total replacement of egg white by faba bean protein concentrate in marshmallow formulation.

The control marshmallow formula (egg-white-based sample) (EWM) was prepared using the following percentages: 2.82% gelatin, 11.27% egg white, 56.34% sugar, and 11.27% glucose syrup. Water was added in two distinct steps: 8.45% for gelatin hydration and 9.86% during the preparation of sugar and glucose syrup (see Figure 1 for details).

A solution of gelatin and cold water was prepared first for hydration. A mixture of sugar, glucose syrup, and water was heated until the sugar was completely dissolved under a hot plate to a temperature of 121 °C. The second step involved adding the hydrated gelatin mass to the hot syrup for 6 min, which caused the temperature to drop to 84 °C. Using a “Kenwood” brand mixer and at maximum speed, the EW was whipped for 8 min, after which the syrup–gelatin mixture was added in a stream at minimum speed for 5 min until the preparation was completely cooled. After molding, the resulting product was covered with starch and cooled for 3 to 4 h at a temperature of 20 °C, then cut into uniform squares. Storage was realized in an airtight container or aluminum bags. In the FPCM formulation, egg white was completely replaced by faba bean protein concentrate (FPC), along with sufficient water to achieve the same moisture content as in the EW formulation.

No adjustments were made to processing parameters when egg white was replaced by FPC. Mixing speed, whipping time, syrup addition rate, gelatin incorporation time, cooling duration, molding conditions, and storage conditions were kept identical for all formulations. This experimental design was chosen to ensure reproducibility and to allow for a direct and valid comparison of the textural and functional properties of marshmallows prepared with different protein sources.

#### 2.4.1. Physico-Chemical Characterization

Water activity, pH, dry matter, and protein content were measured as described in the previous section.

The density at 25 °C was determined by dividing the product’s volume (mL) by its quantity (g) when it was put into a 50 mL measuring cup.

#### 2.4.2. Texture Profile Analysis

Texture parameters were evaluated as described for protein gels. TPA was carried out using a 35 mm diameter cylindrical probe. Marshmallow samples (10 mm height × 20 mm length) were compressed by 50% at a speed of 10 mm/s, and texture parameters were determined using the same cited software.

#### 2.4.3. Microstructure

Utilizing scanning electron microscopy (SEM), the microstructure of marshmallow samples was examined. The samples were adhered to carbon sample holders with double-sided tape. Using a JEOL JFC-1100E ion sputtering apparatus (Fine Coat, JEOL Ltd., Tokyo, Japan), samples were sputter-coated with a 20 nm layer of gold to improve conductivity before imaging. Using a JEOL JSM-7100F Field Emission Scanning Electron Microscope (JEOL Ltd., Tokyo, Japan) in low vacuum (LM) mode and an accelerating voltage of 15 kV, a microstructural investigation was carried out. Images of micrographs were taken at 1000× magnification.

#### 2.4.4. Mid-Infrared Measurements

An attenuated total reflection (ATR) accessory with a grip (Pike Technologies, Inc. Madison, United States) was mounted on an IRTracer-100 Fourier transform spectrometer (Shimadzu, Duisburg, Germany) to record infrared spectra at room temperature (20 °C) between 4000 and 700 cm^−1^ at a resolution of 4 cm^−1^. A horizontal ZnSe crystal with a 45° incidence angle and total reflection (n = 10) served as the basis for the ATR cell. The center of the marshmallow was cut into a slice that was 1.5 cm thick, 5 cm long, and 5 cm deep. It was then crushed for 40 s at 2500 rpm and 20 °C using a Retsch GM 2000, Germany. After that, the ground sample was put on top of the crystal, and pressure was applied to the grip to guarantee that the sample and crystal made good contact. The ZnSe crystal’s spectrum was captured and used as background before each measurement [10].

#### 2.4.5. Front-Face Fluorescence Spectroscopy Measurements

Fluorescence spectra were recorded using a Fluoromax-4 spectrofluorometer (Jobin Yvon, Horiba, NJ, USA). The excitation radiation’s angle of incidence was chosen at 60° to minimize depolarization events, scattered radiation, and reflected light. With a thermostatic cell, the spectrofluorometer’s temperature was controlled by a Haake A25, AC 200 temperature controller from Thermo-Scientific, France. The marshmallow sample was cut in the middle into slices that were 3 cm long, 1 cm wide, and 0.7 cm thick. At 20 °C, the marshmallows’ spectra were recorded while they were placed between two quartz glasses. For every marshmallow, three spectra were produced using various samples. The sample was illuminated in the center by excitation photons (light beam, about 3 mm high and 0.3 mm wide) to keep it from drying out too much. According to Botosoa et al. [11], vitamin A was excited at 250–350 nm, and the emission spectra were recorded at 410 nm. Tryptophan residues were excited at 290 nm, and their emission spectra were obtained at 305–400 nm. During excitation, all spectra were corrected for instrumental aberrations using a rhodamine cuvette in the reference channel.

#### 2.4.6. Principal Component Analysis (PCA)

Principal component analysis (PCA) was used for both FTIR and fluorescence spectral datasets to minimize the dimensionality of the data and to identify the main sources of variability between samples. Before analysis, spectra were pre-processed to reduce instrumental noise and intensity fluctuations. The resulting data matrices (samples × variables) were mean-centered prior to PCA calculation. Score plots (e.g., PC1 vs. PC2) were used to demonstrate clustering and separation of formulations, while loading plots were examined to identify the spectral regions (wavenumbers for FTIR, emission wavelengths for fluorescence) most responsible for discrimination. This multivariate technique made it possible to correlate structural changes with spectral variations in the different marshmallow formulations made with EW protein or with FPC as a substitute.

#### 2.4.7. Hedonic Evaluation

The marshmallow samples were prepared one day in advance, stored, and served at room temperature. A hedonic test was carried out by 60 judges based on a 5-point hedonic scale (1 = very unpleasant; 5 = very pleasant). All portion orders were fully randomized, and the samples were coded using three-digit random numbers. Water was available for washing in between the tests. Evaluation was based on overall appearance, firmness, taste, smell, and overall acceptability. The studied matrix is commercially available and considered a safe product; therefore, no formal authorization from an institutional ethics committee was required. The University of Sfax in Tunisia provided verbal ethical approval. All participants were informed of their rights, the evaluation procedure, and this study’s objectives. Participation was entirely voluntary. Vulnerable groups, such as children or individuals with disabilities, were not included in the test. In accordance with institutional procedures, each panelist verbally gave informed consent to participate in the sensory analysis. All information was kept confidential and was not disclosed without consent. Participants could withdraw from this study at any time without any consequences.

### 2.5. Statistical Analysis

Using Minitab Software version 16, the data were analyzed and compared using Tukey’s test at a significance threshold of α = 5%. Every experiment was performed at least in triplicate (n = 3).

## 3. Results

### 3.1. Physico-Chemical Properties of Egg White and Faba Bean Protein Concentrate

Table 1 summarizes the amount of the various components of EW and FPC. FPC had the lowest water content of approximately 3.91%. The highest content was found in EW (88.37%). Stefanova et al. [12] reported a moisture content around 87.06–88.65% for EW. According to Vogelsang-O’Dwyer et al. [13], the moisture of faba bean protein extract and concentrate could reach 6.11 and 12.2%, respectively. FPC had the lowest water activity value (0.160), and EW had the highest value of about 0.993. EW was found to have a basic pH of 9.27, while the pH of FPC was almost neutral at 6.87.

According to the results in Table 1, proteins were the key component of both ingredients, with the highest values for EW (88.56% DM) compared to FPC (68.79% DM). This finding is consistent with those of Stefanova, Klimenkova, Shakhnazarova, and Mazo [12] and Sebii et al. [14], who demonstrated that EW had a protein content of around 92% DM. Several investigations proved that the protein levels of faba bean protein-rich fractions have ranges between about 50 and 70% DM [15].

### 3.2. Techno-Functional Properties of Egg White and Faba Bean Protein Concentrate

#### 3.2.1. Water Holding Capacity

Table 1 shows that EW had a higher water holding capacity (WHC) than faba bean protein concentrate (approx. 3.435 and 1.749 g/g, respectively). According to Żmudziński et al. [16], faba bean protein had a water holding capacity of about 1.9 g/g at a pH close to neutral.

#### 3.2.2. Surface Properties

##### Foaming Properties

EW’s foaming capacity value (400 ± 10%) is significantly higher than that of FPC (200 ± 10%) (*p* < 0.05). FPC’s foaming ability in this study is close to that determined by Boukid and Castellari [17], who reported that FPC had a foam capacity of 140%.

The foaming stability of EW and FPC was monitored over time (30 and 60 min). The foam capacity of EW declined, while FPC was stable for up to 30 min (FS equal to 96.72 and 100% for EW and FPC, respectively). However, EW had the highest stability after 60 min, around 94.54%, compared to FPC (93.33%).

##### Zeta Potential Measurement

EW had more electronegative charge (−28.43 mV) than FPC (−4.58 mV).

#### 3.2.3. Gelling Capacity

##### Least Gelling Concentration

The LGC was equal to 14% for our faba bean protein concentrate (FPC). According to earlier research on faba bean protein isolate (FPI) gelling properties, the minimal gelling concentration ranges between 12 and 14% [18]. In contrast, Badjona et al. [19] reported that a 10% concentration was sufficient to form a self-supporting gel for protein isolates from peas, soy, and ultrasonically extracted faba bean protein isolate at pH 7. EW proteins required only 10% *w*/*v* to form a gel, which is consistent with the value reported by Adebayo and Oladipo [20].

##### Texture Profile Analysis of Gels

EW forms a very firm gel compared to FPC, with a value of 2552.56 and 746.096 g, respectively (Table 2). The results showed that the EW gel is more cohesive and elastic compared to FPC, with values of 0.693 and 0.910 compared to 0.305 and 0.768.

### 3.3. Characterization of Marshmallows

#### 3.3.1. Physico-Chemical Characterization

The sample prepared with FPC had the lowest moisture content compared to the EW-based formulation (EWM), defined as the control sample (17.337 and 17.598%, respectively). The FPCM was found to have a higher water activity level than that made with EW, with values equal to 0.698 and 0.685, respectively. The control marshmallow had the highest protein content (4.82 and 4.46% for EW and FPC marshmallow, respectively). The EWM was less dense (0.463 g/mL) than the FPCM (0.519 g/mL).

#### 3.3.2. Textural Parameters

According to Table 3, the FPCM had a significantly higher hardness than the EWM (1410.48 versus 1085 g, respectively), which had the highest cohesiveness and adhesiveness values equal to 0.798 and 51.693 for the EWM versus 0.777 and 2.051 for the FPCM. The highest elasticity value was recorded for the FPCM compared to the EWM (88.657 versus 88.524 mm, respectively). The FPCM also had the highest gumminess and chewiness values, attaining 969.830 and 969.912 g.

#### 3.3.3. Microstructure

**Figure 2:**
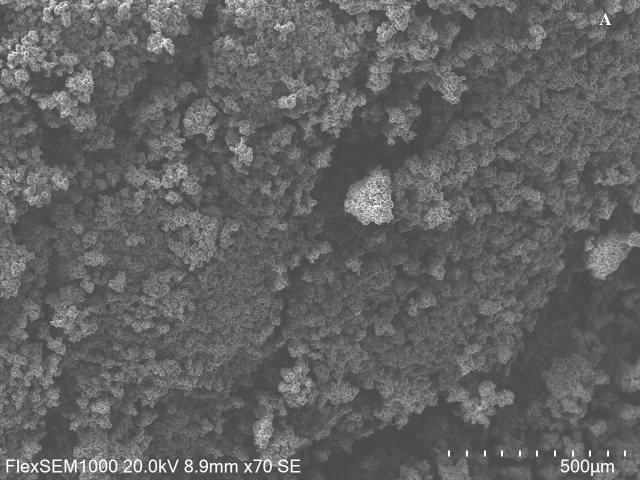

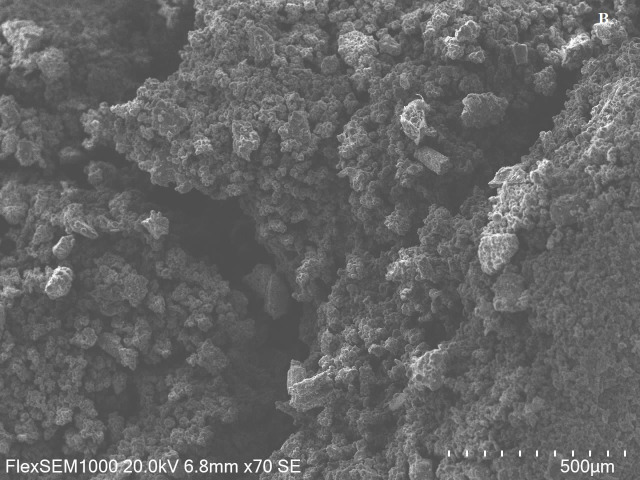
Scanning electron micrographs at 1000-fold magnification: (**A**): Control marshmallows; (**B**): Faba bean protein concentrate marshmallows.

Figure 2 shows the SEM images of the FPCM compared with the EWM. The SEM analysis revealed that despite some differences, the overall foam structure of the FPCM was similar to that of the control, and they had a comparatively stable and airy structure. Although not exactly the same, the microstructure showed that the FPCM structure (Figure 2B) is sufficiently similar to the EW formulation (Figure 2A) in terms of wall development, air cell distribution, gelling behavior, and network formation to support its potential as an acceptable substitute for EW in aerated confectionery. The EWM has a fine and homogeneous pore network with small, evenly distributed air cells. In contrast, the FPCM has thicker walls and larger, slightly irregular air cells.

#### 3.3.4. Mid-Infrared Measurements

**Figure 3:**
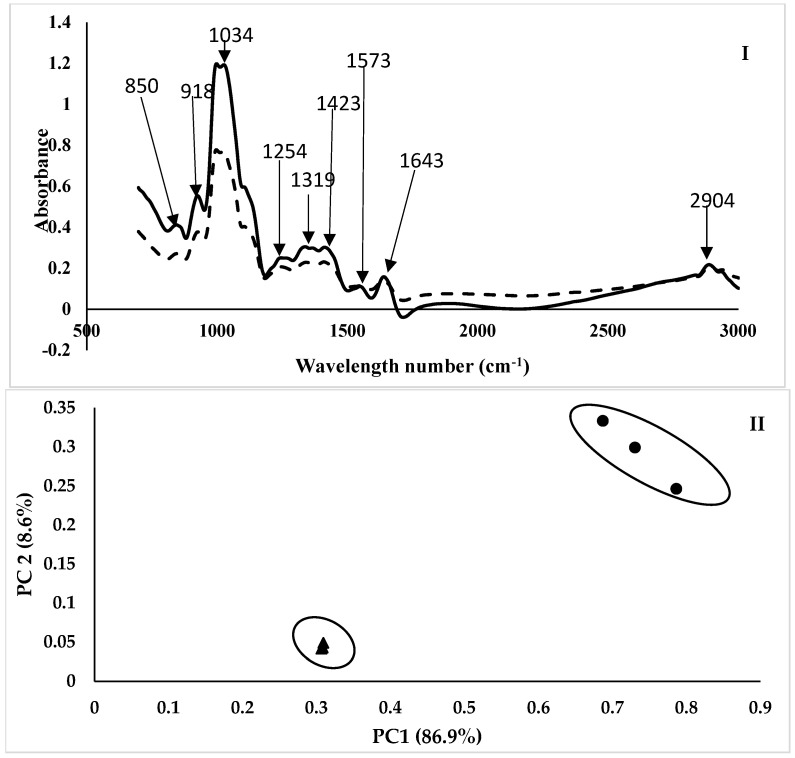
(**I**). MIR spectra of control and faba bean protein concentrate marshmallows 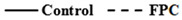. (**II**). Principal component analysis 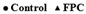.

Several absorption bands relative to the basic valence vibrations of molecular functional groups were discovered in the 500–3000 cm^−1^ spectral region (Figure 3I) [21,22,23]. The 1156–700 cm^−1^ bands were assigned to the C-O and C-C elongation modes [10]. The highest peaks were at 850, 918, and 1034 cm^−1^ in the control sample compared to the FPCM. The control marshmallow had a higher FTIR absorbance at 1254, 1319, and 1423 cm^−1^. Other bands were discovered at 1573, 1557, 1540, 1519, and 1506 cm^−1^ [24]. The EWM exhibited a higher peak at 1643 and 1573 cm^−1^ compared to the FPCM. Spectra from both formulas were analyzed using multivariate statistical techniques, such as principal component analysis. FPCMs were primarily distinguished from the control by the first two main principal components (PCs), which accounted for 86.9% of the total variance (Figure 3II). In fact, both formulas were positioned on the positive side of PC1. A distinct differential of marshmallows based on their formula was noted when PC2 was taken into consideration, accounting for 8.6% of the overall variance.

#### 3.3.5. Fluorescence Measurements

**Figure 4:**
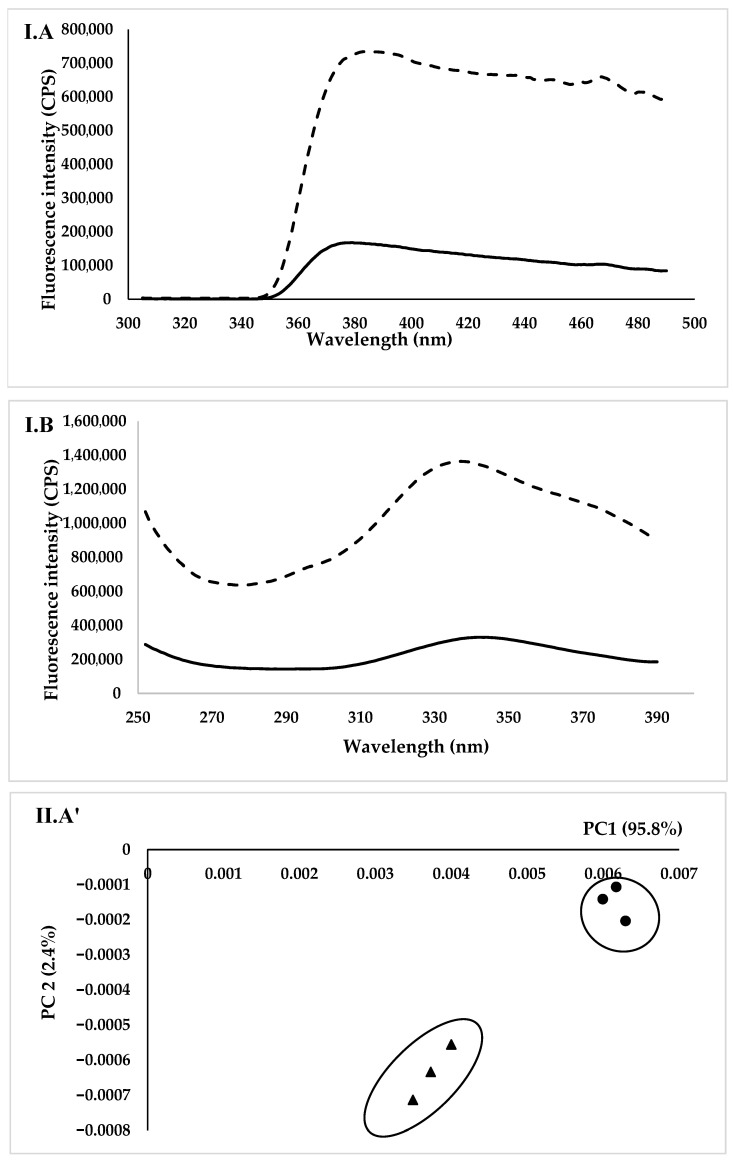

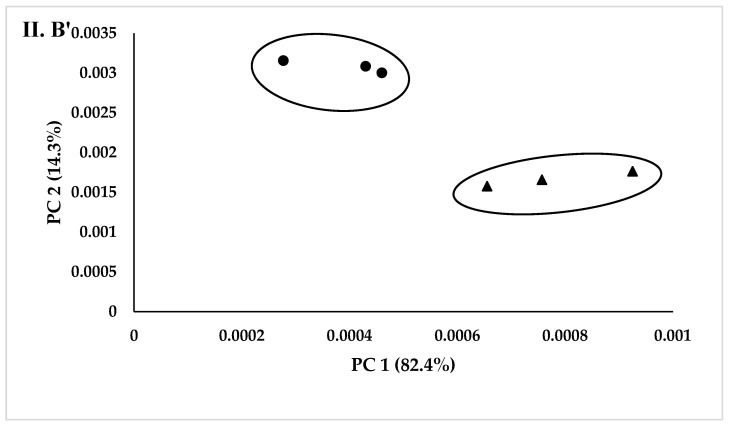
(**I**). Normalized emission fluorescence spectra acquired after excitation of control and faba bean protein concentrate marshmallows. (**A**): Tryptophan emission spectra. (**B**): Vitamin A emission spectra 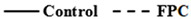. (**II**). Principal component analysis. (**A’**): Tryptophan emission spectra 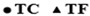. (**B’**): Vitamin A emission spectra 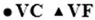.

Figure 4(IA) shows the normalized tryptophan fluorescence spectra of the EWM and FPCM recorded after excitation at 290 nm. Both spectra showed a similar shape with a maximum at 382 nm, which is attributed to the highest emission of tryptophan [11]. The spectra showed that the FPCM had the highest intensity compared to the control, with no shift in emission wavelength.

After excitation at 250–350 nm for vitamin A, the fluorescence spectra of both the control and FPCM showed a maximum located around 338 nm and a shoulder around 280 nm, with a higher emission for the FPCM.

Principal component analysis (PCA) was applied, separately, to tryptophan and vitamin A spectra (Figure 4II). The similarity map of tryptophan spectra demonstrated a clear distinction of marshmallow samples based on their formulas (95.8 and 2.4% of the total variance, for PC1 and PC2, respectively). According to PC1, both samples had positive scores and were positioned on the positive side. Additionally, both samples showed negative scores according to PC2, while control samples exhibited mostly positive scores according to PC1. Regarding vitamin A spectra, a clear discrimination of control and FPC samples was noted in the similarity map (82.4 and 14.3% of the total variance, for PC1 and PC2, respectively). Regarding PC1, both samples had positive scores. In addition, all samples exhibited positive scores according to PC2, while control samples had the most positive scores.

#### 3.3.6. Hedonic Evaluation

**Figure 5:**
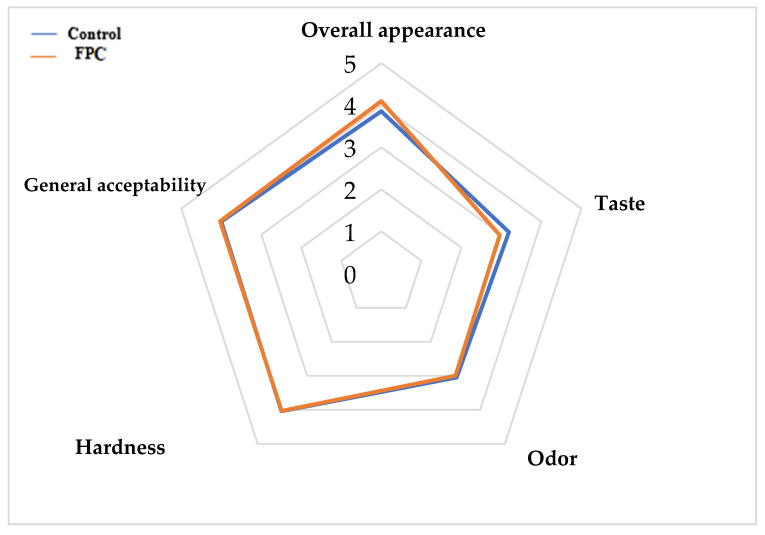
Sensory evaluation of egg white- and faba bean protein concentrate-based marshmallows.

Figure 5 shows a sensory evaluation of the appearance, odor, color, taste, hardness, and overall acceptability of marshmallow samples.

Hedonic evaluation showed that the FPC sample and the control sample are clearly similar in terms of hardness, odor, and general acceptability. The control marshmallow had the highest score for the taste descriptor (rating of 3.19), exceeding that of FPC, while the FPC sample had the highest score in terms of overall appearance (rating of 4.10). These results proved the success of totally replacing EW with FPC in the marshmallow formulation, as evidenced by the comparable general acceptability scores obtained for both formulations (rating of 4.03).

## 4. Discussion

The characterization of the used ingredients is a crucial step that allows us to relate the physico-chemical properties to the observable techno-functional capacities of both ingredients. The difference in moisture content between EW and FPC could be explained by the highly aqueous composition of the egg compared to the faba bean protein concentrate flour. Furthermore, the drying techniques used for FPC could also be the origin of possible variations with recorded moisture contents in the literature. Water activity, combined with the moisture content, increases the risk of microbial growth [14]. As a result, FPC powder, which exhibited the lowest values for both parameters, can be stored for longer periods, making it more suitable for later use.

Proteins were the key component of both ingredients, although a significant difference was observed. The origin of the raw material and the various extraction procedures play a major role in determining the amount of extracted protein and might produce protein extracts with a protein concentration around 70% and isolates with a protein content higher than 80% [25]. Despite its moderate protein concentration, faba bean protein concentrate has shown remarkable potential as an egg substitute in food applications such as baked goods, dressings, and desserts, owing to its multi-functional properties [26]. Based on the physico-chemical characterization, it may be concluded that FPC represents a promising ingredient for replacing EW in food formulations.

The variation in the water holding capacity of EW and FPC could be related to numerous variables, including the proteins’ amino acid composition, structure, solubility, surface hydrophilicity, and extraction method [27]. Indeed, EW proteins such as ovalbumin and ovotransferrin are highly soluble and flexible, which allows them to expose polar and charged amino acid residues that easily form electrostatic contacts and hydrogen bonds with water molecules [28]. The ability of these proteins to bind water is enhanced by their large surface area and limited interaction with non-protein substances. On the other hand, the main component of faba bean proteins is storage globulins, such as legumin and vicilin, which have compact, globular conformations that minimize water binding by limiting surface hydrophilic exposure [6]. Furthermore, the effective WHC of the protein matrix is further reduced by the presence of non-protein substances such as fibers, polyphenols, or starch, in faba protein extracts. These substances may compete with water or form physical barriers [29].

Foaming capacity (FC) is expressed as a volume increase (%) after protein whipping, while its stability is determined by the change in foam volume over time [30,31]. The high protein content of EW, particularly ovalbumin and lysozyme, contributes to its foaming ability. These amphiphilic proteins tend to spontaneously adsorb at gas–liquid interfaces, especially those created by mechanical whipping. This phenomenon stabilizes air bubbles, which in turn produces foam. Lau and Dickinson [32] reported that the foam collapsed due to gravity when the EW protein concentration fell below 0.2%. However, foamability is positively impacted by a high protein concentration (>6%). The primary protein found in legume faba beans is globulin, which constitutes one of the four main classes of plant proteins. Vicilin is a 7S globulin storage protein that is the main protein in faba beans responsible for their foaming ability [33]. Vicilin exhibited increased surface hydrophobicity and solubility at neutral to alkaline pH levels, leading to an improvement in foaming qualities when compared to legumin (11S globulin). These properties allow vicilin to quickly adsorb at the air–water interface, unfold, and create stable films that efficiently capture air bubbles, thereby enhancing foam formation and stability [33].

Concerning the foaming stability of EW and FPC, the differences can be attributed to variations in their chemical composition and protein structure. Proteins, due to their amphiphilic nature, readily adsorb at the air–water interface, making them key contributors to foaming properties in many food systems. Protein–protein interactions, along with interactions between proteins and other components in the matrix, contribute to the formation of a viscoelastic film around air bubbles, which prevents bubble collapse and enhances foam stability [34]. The foaming ability of EW primarily stems from the synergistic action of its various proteins, which not only facilitate efficient foam formation but also maintain foam stability over extended periods [35].

In the food industry, the zeta potential of a molecule is an important characteristic. The electrostatic potential of a material determines its ability to interact with other molecules in suspension through ionic forces [36]. Variations in protein structure, amino acid composition, and the level of surface charge exposure are the main causes of the observed difference in the sample’s electronegativity. Indeed, the zeta potential and the measurement of the isoelectric point allow for the prediction of particle interactions in the construction of complicated systems. In this measurement, solutions were prepared at pHs equal to 9.27 and 6.87 for EW and FPC, respectively. It should be noted that EW’s pH value was much higher than its isoelectric point (4–6.5), while FPC’s pH value was much closer to its isoelectric point (4–5.5) [6], which explains the highest electronegativity of EW compared to FPC. As EW showed the highest FC compared to FPC, this could be strongly related to its highest electronegativity, which ensured molecule repulsion and interaction with water molecules, leading to the formation of foam.

The least gelling concentration (LGC) is the minimum protein concentration needed to create a self-supporting gel that does not slide or flow under gravity when the sample is heated and cooled, and the container is inverted. The gelling capability of proteins is higher when their LGC is lower [33,37].

The difference in the LGC is attributed to the distinct protein compositions and structures of EW and faba bean proteins. EW proteins, particularly ovalbumin, exhibit excellent gelling properties due to their unique structural and functional characteristics. Ovalbumin has a highly organized tertiary structure that unfolds upon mild heating (starting around 60–65 °C), exposing hydrophobic and reactive sulfhydryl groups. This unfolding promotes protein aggregation through disulfide bond formation, hydrophobic interactions, and hydrogen bonding, resulting in the formation of a firm, elastic gel network [38]. In addition, the high solubility of EW proteins in aqueous environments also facilitates even denaturation and network formation. These properties collectively enable EW to form gels at relatively low concentrations (~5–7.5% *w*/*v*) compared to many plant proteins [39].

The texture profile of gels formed from EW proteins is different from gels formed with faba bean proteins, which is largely due to the differences in protein composition. Ovalbumin and ovotransferrin in EW unfold upon slight heating (~60–70 °C) and form solid gels through disulfide bonds and hydrophobic interactions [39,40]. These covalent cross-links lead to a compact, elastic network and contribute to the firmness of the gel. In addition, the proteins are highly soluble, which promotes uniform distribution and efficient network formation during gelation [41].

In contrast, the high functionality of globulins in FPC is demonstrated by their ability to gel when heated in an aqueous solution [42]. FPC consists mainly of vicilin and legumin, storage globulins with few sulfhydryl groups, which means that fewer disulfide bonds are formed during heat-induced gelation. Their gelation largely depends on non-covalent interactions such as hydrogen bonds and hydrophobic effects, which lead to weaker, less cohesive gelation [43]. Oluwajuyitan and Aluko [33] report that common faba bean (Vicia faba) protein lacks sulfur-containing amino acids, especially cysteine and methionine, which are a limiting factor in the amino acid profile and reduce the formation of disulfide bonds during gelation, resulting in a less firm, cohesive, and elastic gel.

Despite legume proteins such as FPC generally exhibiting inferior techno-functional properties compared to EW proteins, they still possess acceptable functionality for use in food formulations, making them a viable alternative in certain applications [44].

After the formulation of marshmallows, physico-chemical characterization was carried out to compare the obtained final products and to discuss the effect of the total substitution of EW with FPC in the marshmallow formula.

The moisture content is considered one of the key factors affecting the acceptability, shelf life, packaging, and storage requirements for compound foods [45]. The recommended moisture content for this type of aerated product is 15–22 g/100 g [46]. The moisture values obtained could be explained by the fact that ovalbumin in EW proteins have strong water-binding properties and form stable foams that efficiently retain moisture in the marshmallow matrix. On the other hand, the lower water-holding capacity of FPC leads to higher moisture loss during processing. In addition, the less stable foam of FPC allows more moisture to evaporate during the heating and drying phase of the marshmallows, which explains why the final product had a lower moisture content.

Water activity is a suitable parameter for assessing the microbiological stability and shelf life of food products, and it indicates the amount of water accessible in the system [47]. According to Palabiyik et al. [48], the water activity of a freshly manufactured marshmallow, which ranges between 0.600 and 0.750, varies depending on its composition and water content. It also decreases over time. Despite having a lower moisture content, marshmallows produced with FPC have been found to have a higher water activity than those made with EW. Both values remain close to the recommended range for shelf-stable marshmallow products. The main causes of these values are the faba bean proteins’ decreased hydrophilicity and lower water holding capacity (WHC). Ovalbumin and other EW proteins form networks that efficiently bind and trap water molecules in the marshmallow matrix, thus reducing water availability [39]. In contrast, faba bean proteins are less water-soluble and more hydrophobic [17]. Consequently, a greater percentage of water remains free and unbound, which increases the aw in FPC-based formulations.

The higher protein content of EW compared to FPC was the reason for the high amount of protein in the EWM compared with the FPCM.

Marshmallow density, which measures the amount of air incorporated during whipping, is the mass of the foam product per unit volume. Higher air content and increased foamability are indicated by lower density values, and these factors have a direct effect on the confectionery product’s mouthfeel, texture, and overall quality [49]. The lower density of the EWM might be linked to the discussed foam capacity, proving that the sample with the highest capacity adsorbs more air and produces a lighter foam.

A product’s texture is a crucial factor in determining its acceptance [45]. The complete substitution of EW in the final product formulation significantly affected the textural properties. Hardness values could be related to the variations in the foaming properties of the used ingredients and the associated density of the final products. In fact, a reduced amount of incorporated air, resulting from the lower foaming capacity and expressed by higher density, led to a harder final product [50].

On the other hand, the hardness of the FPCM was the highest, even though EW proteins exhibited a lower LGC. This could be explained by variations in protein–gelatin interactions and network structuring during foaming and processing. The main gelling agent in marshmallow compositions is gelatin, which cools to form a thermo-reversible polymer network, creating an elastic jelly and acting as both an efficient densifier and a foaming agent [2]. However, during aeration and gelation, the interaction between proteins and gelatin chains also has a significant impact on the final texture.

Egg white proteins interact with gelatin mainly through weak non-covalent interactions such as hydrogen bonding and electrostatic attractions, forming flexible and partially interpenetrated networks that contribute to extensibility and reduced hardness [51]. This results in a more open and extensible network producing softer marshmallows. The legumin and vicilin of FPC are less flexible but more likely to group due to hydrophobic interactions and limited unfolding [43]. These plant proteins tend to form denser and more aggregated structures, which can physically bend the gelatin chain and generate harder protein–gelatin networks with greater structural rigidity. Additionally, during whipping and cooling, FPC proteins may decrease gelatin mobility and accelerate network solidification, resulting in a more compact and rigid structure. In contrast to the softer interactions observed with egg white, faba bean proteins exhibit lower compatibility with gelatin. This lower compatibility arises from structural and physico-chemical differences: faba bean proteins (mainly legumin and vicilin) are larger, more rigid, and more prone to aggregation than the globular and flexible ovalbumin in egg white. Additionally, mismatches in surface charge and hydrophobicity between faba bean proteins and gelatin reduce their ability to form homogeneous networks. Interestingly, this limited compatibility does not weaken the matrix; under high-shear and sugar-rich conditions, the protein aggregates act as reinforcing domains within the gelatin network, preventing phase separation and enhancing mechanical coupling. As a result, marshmallows containing faba bean proteins exhibit higher hardness, demonstrating that, unlike animal proteins, plant proteins can impart unique and sometimes unexpected textural benefits in composite gel systems.

The higher cohesiveness and adhesiveness of the EWM could be explained by the highest WHC of EW, which led to the production of a cohesive and moist surface. Conversely, FPC’s lower water-binding ability allowed for a drier gel surface, which reduces surface adhesion.

Elasticity is a rheological property of viscoelastic food items that makes them resistant to breaking under pressure and stretching forces that could result in deformation. Such a property should be investigated for marshmallow products. Due to gelatin’s capacity to interact with proteins, marshmallows can tolerate external pressure and regain their original shape once the pressure is released [52]. The highest elasticity value of the FPCM compared to the EWM could be explicated by the flexible nature of the FPC–gelatin network that allows the structure to deform under stress and quickly regain its shape and also by the tightly interconnected and less mobile EW–gelatin networks, which lead to a less elastic EWM.

According to Nateghi et al. [53], gumminess is an additional measurable characteristic of semi-solid foods. The results also showed that using FPC in the marshmallow formula led to a rise in both gumminess and chewiness. This effect can be explained by the formation of a denser protein matrix with reduced aeration, which contributes to a gummier mouthfeel and increased chewing resistance.

In the MIR spectra, bands in the 1156–700 cm^−1^ region were assigned to the C–O and C–C stretching (elongation) modes [10]. Peaks at 850, 918, and 1034 cm−^1^ were attributed to a higher content of free, unbound sugars that are spectroscopically accessible through C–O stretching vibrations characteristic of these bands. Although residual carbohydrates may naturally be present in faba bean protein concentrate, stronger protein–sugar interactions formed during thermal processing, particularly through Maillard-type reactions that lessen the intensity of hydroxyl group vibrations, can reduce their contribution to FTIR absorbance by decreasing their intensity of hydroxyl group vibrations [54]. EW proteins also provide a more homogenous and open matrix, which improves the dispersion and molecular mobility of additional sugars (such as sucrose or glucose syrup), thereby enhancing their infrared signal, particularly at 1034 cm^−1^. However, sugars may be chemically or physically entrapped within the denser or more aggregated structure of faba bean proteins, limiting their spectroscopic contribution in the spectral region associated with C–O stretching vibrations [55].

Proteins exhibit characteristic absorption bands in the 1700–1500 cm^−1^ region. Higher intensities at 1254, 1319, and 1423 cm^−1^ in the EWM may be explained by structural and compositional changes between plant and animal proteins. Stronger absorption in the amide III region (1254 and 1319 cm^−1^), corresponding to N–H bending and C–N stretching vibrations, is observed because EW proteins, such as ovalbumin, are highly purified and possess well-defined secondary structures [56]. In addition, the P=O stretching vibration of phosphoproteins, which are present in EW but largely absent in plant protein concentrate, contributes to the band observed at 1254 cm^−1^ [54]. The increased absorbance at 1423 cm^−1^, assigned to CH_3_ bending and symmetric COO stretching, reflects the higher content of aliphatic site chains and free carboxylate groups in native egg proteins. These vibrational modes may be suppressed or overlapped by residual non-protein components (e.g., polysaccharides, phenolic compounds) in FPC. As a result, FPC-based systems may display lower protein purity, increased aggregation or denaturation, and reduced absorbance intensity in these regions [31]. These spectral differences indicate that the control marshmallow maintains a more open and well-defined protein structure, which enhances the FTIR signal of characteristic side chain and amide vibrations.

In previous research, bands at approximately 1682, 1667, 1651, 1646, and 1635 cm^−1^ were attributed to amide I vibrations [21,23]. Amide II bands were observed at 1573, 1557, 1540, 1519, and 1506 cm^−1^ [24]. The higher peak intensities of the EWM at 1643 and 1573 cm−1 compared to the FPCM are strongly related to the higher protein content, greater purity, higher α-helical content, and well-defined globular structure of egg white proteins. Indeed, EW is a nearly pure protein source, with minimal interference from other macromolecules, and it is therefore used in the control marshmallow formulation. In contrast, faba bean protein concentrate (FPC) may exhibit lower total protein content and higher levels of fiber, carbohydrates, or residual antinutritional compounds, such as vicine, convicine, phytic acid, and trypsin inhibitors, depending on the extraction method employed.

In the fluorescence measurements, the FPCM exhibited the highest fluorescence intensity compared to the control, with no shift in emission wavelength, indicating that the tertiary structure of the protein and the microenvironment surrounding tryptophan residues differ between samples. The fluorescence emission of faba bean proteins may be enhanced by tryptophan residues that are more exposed to a hydrophobic environment or are less quenched by nearby molecular groups, such as polar side chains or disulfide bonds [57]. Furthermore, during the heating and whipping steps involved in marshmallow preparation, the unfolding or partial denaturation of legume proteins may further reveal aromatic residues, thereby increasing fluorescence intensity [58]. In contrast, the ovalbumin and ovotransferrin of EW may interact more strongly with other compounds (such as sugars and air interfaces) or maintain more compact conformations (as demonstrated in the preceding sections), which can reduce fluorescence through quenching mechanisms.

Possible explanations for the higher fluorescence intensity of vitamin A in the FPCM compared to the control include differences in the interaction between retinol and the protein matrix. Faba bean proteins may exhibit weaker binding affinity toward vitamin A than EW proteins, such as ovalbumin or lipocalin-type proteins, which are known to bind retinol strongly, reducing its free concentration and diminishing fluorescence intensity [59]. In contrast, the faba bean protein matrix may enhance vitamin A mobility and exposure to hydrophobic regions, leading to an increase in intrinsic fluorescence.

The hedonic evaluation showed that marshmallows prepared with faba bean protein concentrate (FPCM) and those made with egg white (EWM) were similar in terms of hardness, aroma, and overall acceptability, confirming the sensory success of completely replacing egg white with faba bean protein concentrate. This absence of significant differences should be interpreted as a key technological and practical achievement rather than a limitation. It demonstrates that faba bean protein can successfully reproduce the essential foaming and structural functions of egg white in a confectionery matrix without compromising consumer perception. Functional parity between the FPCM and EWM is particularly relevant because it enables the replacement of an animal-derived ingredient with a plant-based alternative while maintaining product quality. In addition, using faba bean protein provides advantages such as suitability for vegan and vegetarian diets, reduced dependence on animal agriculture, and improved sustainability due to the lower environmental footprint of legume proteins. Furthermore, egg white is a major food allergen, whereas faba bean protein is less commonly associated with severe allergic reactions, potentially offering safer options for consumers, although proper labeling remains essential. Taken together, the comparable hedonic response confirms that faba bean protein is a viable and scientifically justified substitute for egg white in marshmallows, delivering equivalent sensory quality while providing meaningful nutritional, ethical, and sustainability-related benefits.

## 5. Conclusions

The present study describes the results of the total substitution of egg white by faba bean protein concentrate (FPC) in a marshmallow formulation. The foaming ability of FPC did not exceed that of EW, with a comparable foaming stability, which was associated with the highest electronegativity of EW measured by zeta potential. FPC gelified at a higher LGC compared to EW, and the formed gel was less hard, related to the differences in protein composition.

The physico-chemical properties of the final products indicated that the FPCM had a higher density and lower moisture and water activity within the range of this type of product, which were attributed to the foaming properties, the low WHC of FPC, and the nature of interactions between proteins and gelatin. The texture profile analysis of marshmallows showed that the hardness and the elasticity of the FPCM were higher, while cohesiveness and adhesiveness were lower than those of the control sample. The hardness of the FPCM was related to the nature of the interaction between protein and gelatin, which is stronger in the case of FPC than EW, which reduces gelatin mobility, creating a more compact and rigid structure. The greater elasticity of the FPCM can be explained by the flexible nature of the FPC–gelatin network. The adhesivity of the FPCM is the result of the lower water holding capacity of FPC, which leads to higher moisture loss during processing, producing a moist surface. SEM analysis proved that the overall foam structure of the FPC marshmallows was similar to that of the control formulation, with a comparatively stable and airy structure. Structural analysis by FTIR revealed that stronger protein–sugar interactions occurred during the thermal processing of the FPC marshmallow, particularly through Maillard reactions that reduce the intensity of hydroxyl group vibrations, and that the sugars may be chemically or physically trapped by the denser or more aggregated structure of faba bean proteins. The highest tryptophan and vitamin A intensity in the FPC marshmallow could be explained by the unfolding or partial denaturation of the faba bean proteins, releasing more aromatic residues and poorer binding associations with vitamin A, compared to EW, which enhances fluorescence.

The hedonic evaluation showed that FPC and the control sample were clearly similar in terms of hardness, odor, and general acceptability, demonstrating the successful complete replacement of EW with FPC.

## Figures and Tables

**Table 1:** Physico-chemical and techno-functional characterizations of egg white and faba bean concentrate.

Parameter	EW	FPC
**Moisture (%)**	88.370 ± 0.06 a	3.910 ± 0.09 b
**Proteins (%DM)**	88.560 ± 0.60 b	68.790 ± 0.03 a
**Water activity**	0.993 ± 0.002 a	0.160 ± 0.002 b
**pH**	9.270 ± 0.017 a	6.870 ± 0.021 b
**Water holding capacity (g/g)**	3.435 ± 0.04 a	1.749 ± 0,06 b
**Foaming capacity (%)**	400.000 ± 10.00 b	200.000 ± 10.00 a
**Foaming stability (%) (30 min)**	96.720 ± 0.01 a	100.000 ± 0.01 b
**Foaming stability (%) (60 min)**	94.540 ± 0.01 b	93.330 ± 0.01 a
**Zeta potential (mV)**	−28.430 ± 0.09 a	−4.580 ± 0.03 b
**Least gelation concentration (%)**	10.000	14.000

EW: egg white; FPC: faba bean protein concentrate; DM: dry matter. The results are represented as the mean ± standard deviation (n = 3). a,b,: Letters that differ on the same line indicate a significant difference between the mean values at a 5% level of significance.

**Table 2:** Texture profile analysis of egg white and faba bean concentrate gels.

	EW	FPC
**Hardness (g)**	2552.564 ± 7.803 a	746.096 ± 2.248 b
**Cohesion**	0.693 ± 0.001 a	0.305 ± 0.015 b
**Elasticity index**	0.910 ± 0.003 a	0.768 ± 0.004 b
**Chewiness (g)**	1609.723 ± 0.214 a	174.765 ± 0.207 b

EW: egg white; FPC: faba bean protein concentrate. a,b: Letters that differ on the same line indicate a significant difference between the mean values at a 5% level of significance.

**Table 3:** Physico-chemical and texture profile analysis of egg white and faba bean concentrate marshmallows.

	EWM	FPCM
**Moisture (%)**	17.598 ± 0.050 a	17.337 ± 0.070 b
**Proteins (%)**	4.820 ± 0.020 a	4.460 ± 0.010 b
**Water activity**	0.685 ± 0.002 b	0.698 ± 0.001 a
**Density (g/mL)**	0.463 ± 0,010 b	0.519 ± 0,030 a
**Texture profile analysis**		
**Hardness (g)**	1294.900 ± 4.749 b	1410.484 ± 1.162 a
**Adhesiveness (g)**	51.693 ± 0.049 b	2.051 ± 0.074 b
**Cohesion**	0.798 ± 0.039 a	0.777 ± 0.001 b
**Elasticity (mm)**	88.524 ± 0.010 a	88.657 ± 0.044 a
**Gummy**	880.013 ± 0.470 b	969.830 ± 0.720 a
**Chewiness (g)**	873.400 ± 3.920 b	969.912 ± 9.620 a

EWM: egg white marshmallow; FPCM: faba bean protein concentrate marshmallow. a,b: Letters that differ on the same line indicate a significant difference between the mean values at a 5% level of significance.

## Data Availability

The original contributions presented in this study are included in the article. Further inquiries can be directed to the corresponding author.
